# Achieving Verifiable Decision Tree Prediction on Hybrid Blockchains

**DOI:** 10.3390/e25071058

**Published:** 2023-07-13

**Authors:** Moxuan Fu, Chuan Zhang, Chenfei Hu, Tong Wu, Jinyang Dong, Liehuang Zhu

**Affiliations:** 1 School of Cyberspace Science and Technology, Beijing Institute of Technology, Beijing 100081, China; 2 Guangdong Provincial Key Laboratory of Novel Security Intelligence Technologies, Shenzhen 518055, China; 3 Defense Innovation Institute, Chinese Academy of Military Sciences, Beijing 100864, China

**Keywords:** verifiable, machine learning, decision tree, Merkle tree, authenticated data structure, blockchain

## Abstract

Machine learning has become increasingly popular in academic and industrial communities and has been widely implemented in various online applications due to its powerful ability to analyze and use data. Among all the machine learning models, decision tree models stand out due to their great interpretability and simplicity, and have been implemented in cloud computing services for various purposes. Despite its great success, the integrity issue of online decision tree prediction is a growing concern. The correctness and consistency of decision tree predictions in cloud computing systems need more security guarantees since verifying the correctness of the model prediction remains challenging. Meanwhile, blockchain has a promising prospect in two-party machine learning services as the immutable and traceable characteristics satisfy the verifiable settings in machine learning services. In this paper, we initiate the study of decision tree prediction services on blockchain systems and propose VDT, a Verifiable Decision Tree prediction scheme for decision tree prediction. Specifically, by leveraging the Merkle tree and hash function, the scheme allows the service provider to generate a verification proof to convince the client that the output of the decision tree prediction is correctly computed on a particular data sample. It is further extended to an update method for a verifiable decision tree to modify the decision tree model efficiently. We prove the security of the proposed VDT schemes and evaluate their performance using real datasets. Experimental evaluations show that our scheme requires less than one second to produce verifiable proof.

## 1. Introduction

With the vigorous development of artificial intelligence, machine learning, the core technology and implementation means of artificial intelligence, has been deployed by many applications since it can extract features and capture relations from data to provide a generalization ability. On the one hand, with the expansion of the research field, machine learning has more application scenarios, such as image classification and recognition [1], natural language processing [2], emotion analysis [3], and disease detection [4]. On the other hand, the blossoming of cloud computing systems [5] enables cloud platforms to undertake large-scale computation tasks of machine learning services to provide efficient machine learning tasks for clients. Thus, cloud platforms have launched the functionality of Machine Learning as a Service (MLaaS) [6]. MLaaS enables service requesters to complete the construction of customized machine learning models through their private data and desired training models, providing a broader and more effective prediction service. Consider a typical application of MLaaS, where a client wishes to evaluate the data under a cloud’s machine learning model. During the prediction phase, the cloud server conducts the prediction service and returns the result to the client.

Meanwhile, due to their high scalability and efficiency, blockchains with hybrid architecture [7,8] have increasingly received consideration. These systems combine the characteristics of blockchains and off-chain services. On-chain consensus makes the system more secure [9], while off-chain computing service increases the efficiency compared to blockchain systems. The blockchain provides an open and transparent platform where transactions and storage are executed in private and immutable manners. Off-chain cloud servers can undertake computing services to solve the problem of the slow computing speed on the blockchain. Due to the immutability and verifiability of blockchain, using a hybrid blockchain system to solve the problems of integrity and verifiability in machine learning services is a promising method.

Despite the enticing benefits of MLaaS, security concerns and privacy issues gradually emerge [10,11,12]. A typical machine learning service for inference on the cloud is as follows. The model owner, e.g., an enterprise with an online cloud server, owns or stores a well-trained machine learning model. The service requester uploads the data sample to the cloud server. In the prediction phase, the cloud server executes the machine learning inference algorithm on the input data to calculate the result. In this scenario, data privacy and model privacy shall be protected since they are the private property of the service requester and the model owner [13]. Meanwhile, the service requester shall refrain from acquiring any information on the machine learning model during the inference phase. Moreover, the model owner may fail to execute the prediction services correctly due to calculation failures or malicious external attacks.

Therefore, machine learning services’ security and integrity threats are increasingly receiving attention. In a cloud computing system, verifying the correctness of machine learning predictions while maintaining privacy is difficult. Thus, ensuring the correctness of prediction results is vital for machine learning prediction services on cloud platforms, which is called the verifiability of machine learning. In cloud setting environments, selfish cloud servers are incentivized to return incorrect inference results for subjective reasons to reduce computational overhead [14]. An opportunistic method for machine learning classification services is to return forged or random classification labels without running the classification algorithms. Therefore, the property of result verifiability is vital for cloud service scenarios. However, creating a solution to ensure machine learning models’ integrity and verifiability takes time and effort. In addition, to meet the needs of machine learning services in more scenarios, the cloud server may update the machine learning model [15]. Thus, it is also essential to efficiently verify whether the cloud server updates the machine learning model while maintaining the verifiability of machine learning prediction. It remains challenging to update the machine learning model on the cloud server.

To address the issues above, researchers focus on designing verifiable machine learning approaches on training and inference processes over various models, e.g., linear regression [16], support vector machine [17], deep neural network [18], and convolutional neural network [19,20,21]. Several verifiable mechanisms have been proposed by [17,22] based on homomorphic encryption methods to verify the correctness of inference results. Niu et al. [17] designed MVP, a verifiable privacy-preserving machine learning prediction service system. The system guarantees the verifiability and privacy-preserving of the SVM machine learning prediction service. Li et al. [22] implemented VPMLP based on OU homomorphic encryption, which performed the prediction of linear regression models in a privacy-preserving manner and designed a verifiable inference scheme for verification. Hu et al. [23] proposed an efficient privacy-preserving outsourced support vector machine scheme (EPoSVM). The authors designed eight secure computation protocols to allow the cloud server to efficiently execute basic integer and floating-point computations in support vector machines. From the above review, however, existing studies only focus on designing the security and privacy of the prediction over a specific machine learning model, which cannot be directly applied to solve the verifiability and privacy-preserving problems of the decision tree model predictions. Additionally, existing studies focus on cloud servers as machine learning service providers and rarely consider cloud servers as proxy machine learning services. However, it is tough to propose a generalized verification scheme for the prediction task of most machine learning models. Researchers need to implement specific verification algorithms for models based on studying the characteristics of different machine learning models.

Because of their great explainability and interpretability, decision tree models stand out among all the machine learning models. Therefore, our study selects the decision tree model as the specific machine learning model. Researchers proposed a few schemes to construct verifiable decision tree prediction to verify the integrity of the decision tree model. Zhang et al. [24] implemented zero-knowledge proof for the decision tree model and proposed zkDT. zkDT exploits the unique properties of the tree structure and constructs commitments from it. zkDT presents efficient protocols to verify decision tree prediction and prediction accuracy by combining zero-knowledge proofs with cryptographic commitments. Wang et al. [25] proposed a specific non-interactive zero-knowledge (NIZK) based on verifiable proof for a private decision tree prediction scheme. The NIZK protocol can prove the correctness of the public verifiable private decision tree prediction results without leaking extra information. However, we have noticed that most system applications are computationally expensive zero-knowledge proof, which limits the practical deployment application of verifiable decision trees. Thus, we aim to discover an efficient way to verify the integrity of the decision tree scheme using a lightweight cryptographic method.

In this paper, we propose a general verifiable decision tree scheme for decision tree prediction in cloud service scenarios to tackle the aforementioned security challenges. Specifically, we construct a verifiable decision tree scheme by leveraging the Merkle tree and hash function. Using the blockchain, the client can effectively verify the correctness of the results. Our VDT scheme ensures the integrity of machine learning predictions and detects errors in machine learning predictions with a 100% probability. We also extend our techniques with minimal changes to support minor updates of the decision tree model. The concrete contributions of the article are summarized as follows:

(1) We proposed a verifiable decision tree scheme to verify the decision tree prediction results. Merkle tree, hash function, and commitments are used to generate the verification proof efficiently.
(2) We further proposed an efficient decision tree update algorithm to support minor model updates. Specifically, the algorithm generates related parameters and proofs of the changes between the prior and current models. It efficiently updates the model without losing the verifiable property of the decision tree.
(3) Taking advantage of the immutable and traceable nature of the blockchain, we employ the blockchain combined with a cloud server to verify the calculation results and guarantee correctness. It can not only realize a simpler verification algorithm, but also acknowledge the service functions of off-chain storage and calculation fairness, which is a promising application scenario in cloud services.
(4) We theoretically analyze the scheme’s correctness and security. The analysis shows that our scheme can perform correctly. We implement and evaluate the proposed scheme using different real-world datasets and decision tree models. The experimental results show that our scheme is practical.

The rest of the paper is organized as follows. Section 2 gives the preliminaries of the decision tree and cryptographic primitives. Section 3 introduces our system model, workflow and security requirements. Section 4 presents our concrete verifiable decision tree scheme. Section 5 provide an update method for decision tree changes. Section 6 analyzes the security of our proposed scheme. Section 7 shows the experimental results of our scheme. Section 8 surveys the related work. Finally, in Section 9, we conclude our work.

## 2. Preliminaries

In this section, we briefly introduce the basic information and primitives that are used in our work.

### 2.1. Decision Tree Classification

Decision tree [26] is one of the most commonly used supervised machine learning algorithms, which models and predicts in a tree structure. Since a lot of data classification work in industrial scenarios attaches great importance to the interpretability of machine learning models, decision tree-based classifiers have been widely used in applications such as disease diagnosing, automated trading and recommendation systems. For example, Adebiyi et al. [27] proposed an ICA learning approach for machine learning diagnosis by meticulously designing a decision tree model.

Our article focuses on binary decision trees for classification tasks for simplicity. A decision tree is constructed by a set of nodes, which can be categorized into three types: root, internal, and leaf nodes. In decision tree *T*, the root node and each internal node contain a comparison of a specific attribute, each tree branch denotes a decision, and each leaf node represents the final classification of the data sample. A typical workflow of decision tree classification is to compare the attributes of the data with the threshold of each internal node in *T* from the root node to the leaf node. Therefore, the prediction path from the root node to the leaf node represents the whole decision-making process and determines the final classification.

More formally, Algorithm 1 shows the algorithm of decision tree prediction. The data sample **a** represents a *d*-dimensional vector corresponding to all *d* attributes used in the decision tree. In *T*, each internal node $v_{i}$ has two children, v.left and v.right, a threshold v.thr for decision comparison, and an associate attribute index v.in, which determines the specific attribute used in the comparison. Each leaf node $l_{i}$ possesses the final classification result $c_{i}.class$. The algorithm starts from the root of the decision tree T.root. For each internal node *v*, it compares the value of attribute a[v.in] corresponding to the index v.in with the threshold v.thr. *v* changes to its left child v.left if a[v.in]<v.thr and its right child otherwise.   
**Algorithm 1:** Decision Tree Prediction**Input:** Decision Tree *T*, data sample a**Output:** Classification result $y_{a}$ 1: v:=T.root 2: **for** *v* is not a leaf node of decision tree *T* **do** 3:  **if** a[v.in]<v.thr **then** 4:   v:=v.left 5:  **else** 6:   v:=v.right 7:  **end if** 8: **end for** 9: **return** $y_{a}:=v.class$

### 2.2. Cryptographic Primitives

In this section, we introduce some cryptographic primitives on which our proposed VDT schemes build.

**Hash Function:** A hash function maps a message *m* to a message digest h(m) with a certain length. We use two main properties of the hash function in our scheme: (1) irreversible, i.e., given a message digest *h*, it is computationally infeasible to find a message *m* such that h(m)=h; (2) collision-resistance, i.e., it is computationally infeasible to find two messages $m_{1}$ and $m_{2}$ such that $h\left(m_{1}\right)=h\left(m_{2}\right)$. In our VDT scheme, the hash function is used to calculate the hash value of the decision tree and the hash value is further used in constructing the verification proof. The irreversibility of the hash function guarantees the privacy protection of the decision tree model during the construction of verification proof, while the collision-resistance of the hash value guarantees the integrity of the prediction result.

**Figure 1:**
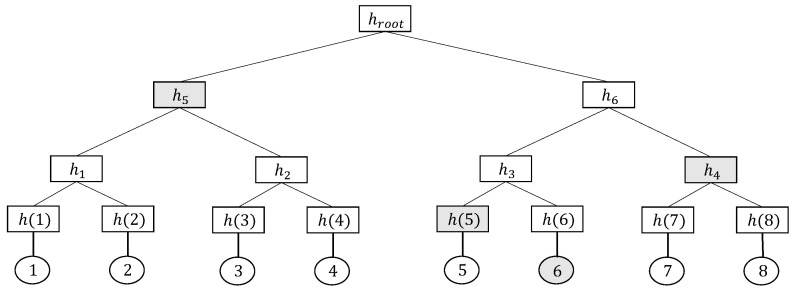
A toy example of a Merkle tree. The shaded nodes make up the verification proof of the Merkle tree.

**Merkle Tree:** A Merkle tree [28] is a type of authenticated data structure for data verification. Figure 1 shows an example of a Merkle tree. Each leaf node holds a digest of stored data, and each non-leaf node represents the tree structure for storing data. First, the hash value of the stored data is calculated when constructing a Merkle tree, which denotes the hash value of the leaf node. Each non-leaf node derives a hash value from its children. For example, in Figure 1, the hash value of non-leaf node $h_{5}$ is calculated by $h_{5}=h_{1}\left|\right|h_{2}$, where the symbol ‘||’ denotes concatenation. The hash value of the root node, which is called the root hash, is stored and published for data consistency checks. The Merkle tree calculates the current hash value of the root node layer by layer and then compares it with the root hash to check the consistency of the stored data. When the two hash value match, the consistency check passes. Otherwise, it fails. Thus, the Merkle tree can verify the stored data in logarithmic complexity. For example, to verify the existence of data whose value is 6, the verification proof of the Merkle tree consists of $h\left(5\right),h_{4},h_{5}$ (shaded in Figure 1). The verifier reconstructs the root hash using the verification proof and the data value and compares it to the public Merkle tree root hash. The existence of data is verified if the two root hash value match. Compared to other storage structures, tree storage structures have lower verification complexity and are more conducive to the construction of our scheme. Section 4.1 provides specific instructions for using Merkle trees.

**Blockchain:** Blockchain [29] is a shared, public distributed ledger consisting of many blocks that store transaction data connected in a chain structure in a traceable and immutable way. It is the underlying technology and architecture of virtual currencies such as Bitcoin. In Bitcoin, each block comprises the block header and the block body. The block header describes the information of the block, which stores the hash value of the parent block, time stamp, nonce, difficulty, and Merkle tree root hash of the current block. The block body contains the transaction information of the data and is responsible for the data storage on the blockchain. In our scheme, the blockchain provides a secure and efficient storage environment that fits our scheme’s verification process.

**Smart Contract:** Nick Szabo [30] first proposed the conception of smart contracts in 1995. A smart contract is a computer protocol that forms a relationship and reaches a consensus among individual users, institutions, and property in an information-based manner and is a computer program that can automatically execute contracts. Ethereum brings smart contracts to the blockchain for the first time. Through transactions, users can deploy or execute smart contracts to implement complex programming operations on the blockchain. On the premise of determining the contract content and contract trigger conditions, the smart contract can automatically execute the contract content when the contract conditions are triggered. The execution results are verified by Ethereum nodes and stored on the blockchain. In our scheme, the interaction between blockchain and other entities is achieved through smart contracts. It can return or update the on-chain hash value storage of the decision tree.

## 3. System Model and Security Requirement

In this section, we first provide the system model and workflow and then describe the security requirements.

### 3.1. System Model and Workflow

**Figure 2:**
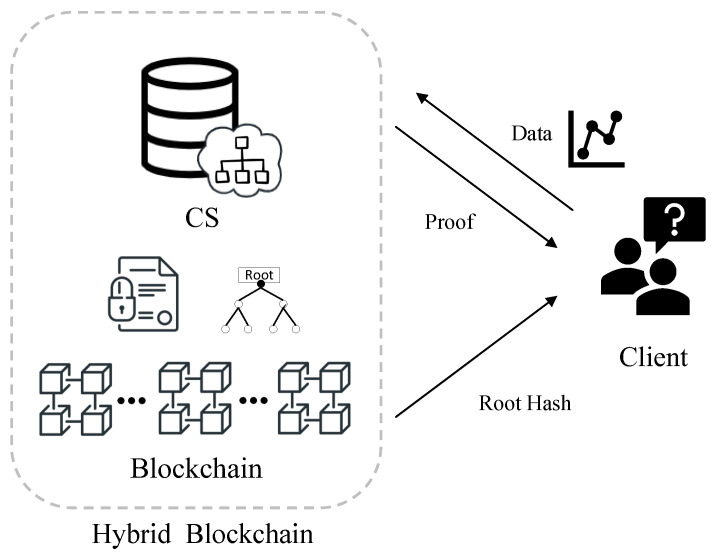
System model of our VDT scheme.

As shown in Figure 2, our verifiable decision tree scheme contains two entities: **Cloud Server (CS)** and **Client**. We identify a workflow for decision tree prediction and verification as follows.

**Cloud Server (CS)**. CS holds a decision tree model, constructs a verifiable decision tree using the hash function and Merkle tree structure, and stores it on the blockchain. CS executes the prediction algorithm with the client’s input data and returns the classification result to the clients. The hybrid blockchain system can efficiently generate the attached verification proof using the prediction path by introducing the blockchain.

**Client**. The client sends its data to the CS and receives the classification result and the verification proof. The client then reconstructs the decision tree’s root hash using the verification proof and compares it to the root hash sent by the blockchain. Finally, the client accepts the classification result if the two hash values match.

### 3.2. Security Requirements

In our system, the client who hopes to obtain the correct classification results over its data is regarded as fully trusted. We consider the CS to be malicious. It may not correctly execute the content of the scheme for some reason. For example, the CS may intentionally forge or modify the result to save computing power or unintentionally performs errors due to the unstable network environment. To detect potentially malicious behavior of the CS with no erroneous judgment and ensure the integrity of machine learning predictions, the client needs to narrow down the following two security criteria:

- **Soundness**: The client can detect CS’s malicious behavior when CS mistakenly executes the scheme and generates modified or forged certificates.
- **Completeness**: When the CS correctly executes the scheme, the proof satisfies the verification of the classification result, and the client confirms the correctness of the returned result.

Regarding the blockchain, we assume that the CS cannot gain any advantage by attacking the consensus protocol. For the smart contract proposed in our scheme, we use a formal verification method to ensure the integrity of the smart contract, which is discussed in Section 6.

### 3.3. Problem Statement

With the above system model, workflow, and security requirements, the problem we study in this paper is how to design a verifiable decision tree scheme that can efficiently support the integrity of the prediction. Since the integrity of machine learning is orthogonal to the privacy-preserving problem in machine learning, our scheme can apply encryption methods to provide privacy-preserving properties.

## 4. Detailed Scheme of Verifiable Decision Tree

In this section, our verifiable decision tree scheme is introduced in detail, containing the basic VDT scheme and the update algorithm. For ease of understanding, the notations used in our scheme are summarized in Table 1.

Our VDT scheme contains four phases, i.e., *Model Initialization*, *Model Prediction*, *Proof Construction*, and *Result verification*. Without loss of generality, we consider binary decision trees as our specific decision tree model for classification problems. Our scheme can be generalized naturally to multivariate decision trees, random forests, and regression trees with minor modifications.

### 4.1. Model Initialization

In the model initialization phase, the CS constructs the verifiable decision tree. Inspired by the Merkle tree generate process, CS selects a specific hash function *H* to construct an authenticated data structure (ADS) based on the tree structure of the decision tree. For binary decision trees, each non-leaf node has two children. As mentioned in Section 2.1, we define the decision branch of the node points to its left child node if the data sample’s attribute is smaller than the decision node’s threshold, otherwise to its right child node. In this tree-shaped ADS, each node not only stores all the information of the original decision tree, but also stores the hash value of the node itself. More specifically, each internal node $v_{i}$ includes the identification number of the node, the associate attribute index and the threshold of the node, the pointer to its child node to maintain the tree structure, and the node’s hash value calculated from its children, recorded as:

$$v_{i}=<id_{i},v_{i}.in,v_{i}.thr,v_{i}.left,v_{i}.right,hash_{v_{i}}>.$$

For each leaf node $l_{j}$, it includes the identification number of the node, the final decision classification result, and the node’s hash, recorded as:

$$l_{j}=<id_{j},l_{j}.class,hash_{l_{j}}>,$$

where the computation of the leaf node’s hash value includes the identification number and the classification result:

$$hash_{l_{j}}=H\left(id_{j},l_{j}.class\right).$$

**Figure 3:**
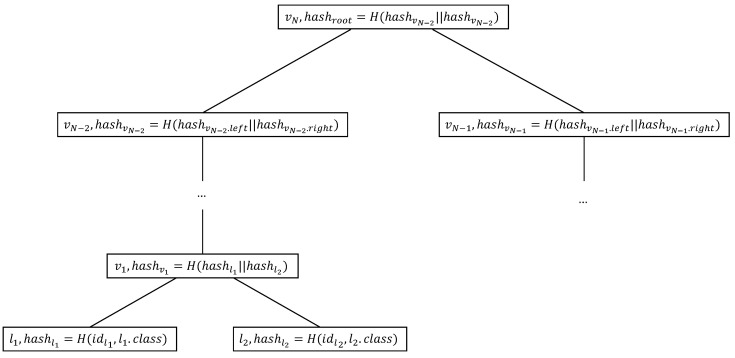
The construction of ADS. Each leaf node contains the identification number, classification result and hash value. Each non-leaf node consists of the identification number, threshold and attribute index, pointers to its children and the hash value.

Figure 3 denotes the construction of ADS. The construction of ADS starts from the hash value calculation of the leaf nodes. Each internal node stores a hash value derived from its two child nodes, calculates hash value calculation from the leaf nodes to the tree root, and finally obtains the hash value of the root node. After the construction of the ADS, CS stores the tree-shaped ADS and the root hash of ADS on the blockchain. Specifically, the root hash of the ADS is public. The storage on the blockchain enables clients to confirm that the CS has a decision tree model, thereby continuing the process of decision tree prediction.

### 4.2. Model Prediction

In this phase, the client sends the input data of the prediction service to the CS. The CS executes the decision tree prediction service and returns the prediction results and related proofs. Due to the classification method of the decision tree, CS obtains not only the inference result, but also a prediction path from the root node to the leaf node. Algorithm 2 describes the detailed procedure of the decision tree prediction service executed by the CS. The algorithm takes the verifiable decision tree *T* and the data sample a as input and outputs the prediction result $y_{a}$ and the prediction path path. CS first initializes the prediction path and current node (line 1). Then, to prove that CS has not changed the decision tree model, the running model is consistent with the model stored on the blockchain. CS commits the ADS with a random number *r* (line 2). More specifically, CS computes hashes from the leaf node to the ADS root with a random number *r*, which is added to protect the privacy of the decision tree model. Then, CS starts the prediction from the decision tree’s root node and determines the decision branches and the prediction path by comparing attributes and thresholds (lines 3–10). When the current decision node *v* is a non-leaf node, CS finds the associate attribute index v.in and the decision threshold v.thr of the current node and compares the threshold v.thr with the value of the associate attribute of the data sample a[v.in]. The current node moves to its left children if a[v.in]<v.thr and otherwise moves to its right children. Meanwhile, the decision branches of all nodes are recorded as the prediction path path. When reaching the leaf node of the decision tree, CS obtains the classification result $y_{a}$ and the prediction path path (lines 11–13), which are sent to the client and construct the verification proof, respectively. Our validation scheme is built on a decision tree and does not affect the process of comparing and making decisions among nodes in the decision tree prediction. Thus, our VDT scheme provides a reliable guarantee for decision tree prediction without affecting prediction accuracy. However, to validate the prediction path’s correctness, the client must check the comparison between the thresholds and the attributes. We have found that this problem can be effectively and quickly solved through auxiliary proofs. Thus, in our VDT scheme, CS leverages techniques in [31] to generate an auxiliary proof for prediction path aux for comparison operations in the verification process and sends aux to the client.   
**Algorithm 2:** Model Prediction**Input:** Verifiable Decision Tree *T*, data sample a**Output:** Prediction Result $y_{a}$, Prediction Path path  1: Initialize path as a list path:=[T.root]      Initialize current node v:=T.root  2: $com_{T}\leftarrow$ Commit(T,r): the CS commits the verifiable decision tree by computing       hashes of the root node from the leaf nodes with a random number *r*  3: **for** *v* is not a leaf node of decision tree *T* **do**  4:  **if** a[v.in]<v.thr  5:   *v* points to the left children v:=v.left  6:  **else**  7:   *v* points to the right children v:=v.right  8:  **end if**  9:  path.append(v)10: **end for**11: **if ***v* is a leaf node of decision tree *T* **then**12:  **return** $y_{a}=v.class$, path, aux13: **end If**

### 4.3. Proof Construction

Following the algorithm to obtain the classification result, we further present our scheme to prove the correctness of the prediction. A naive approach is to directly send the prediction path with the whole construction of ADS to the client. However, recent research shows that the client can infer sensitive information or reconstruct the decision tree models with several prediction paths obtained. Thus, this approach is impractical since it would cause model privacy leakage. To protect the privacy of the decision tree model, we propose the verification proof in a way that does not return plaintext information of the prediction path. We divide the whole verification process into three parts: (1) Determine whether the decision tree has changed; (2) check the validity of the prediction path path; (3) verify that the classification result $y_{a}$ is calculated from the data sample **a**. The commitment $com_{T}$ constructed in Algorithm 2 ensures the correctness of the first part. Then, we construct the relative proofs for verifying the prediction path and data sample using the Merkle-tree-based ADS. We will analyze the correctness of the proofs in Section 6.

**Proof for the prediction path.** After the CS obtains the predicted path, it uses Algorithm 3 to generate a verifiable proof for the prediction. The Proof Generation algorithm takes the Merkle-tree-based ADS *T* and prediction path path as input and outputs the verification proof π. The algorithm is similar to the verification method in a Merkle tree. The verification proof for prediction includes information on the prediction path path, which denotes the whole procedure in the inference process. In addition, due to the tree structure of our ADS, the hash values of all the siblings of the node contained in path should be included (lines 3–5). By executing Algorithms 2 and 3, the CS obtains the classification result $y_{a}$ of the input data, the predicted path path, and the related proof π based on the ADS structure. The classification result $y_{a}$, the commitment $com_{T}$, and the related proof π are sent to the client.
**Algorithm 3:** Proof Generation**Input:** Verifiable Decision Tree *T*, Prediction Path path**Onput:** Verification Proof π  1: Initialize list $\pi :=[hash_{path[0]}]$      Initialize node *v*, vp, $v^{\prime }$  2: **while**  the length of prediction path len(path)≠0 **do**  3:  aux  4:  v=path[0]**          **vp=path[1]  5:  find the other child node $v^{\prime }$ of the parent node of vp  6:  $\pi .append(hash\left(v^{\prime }\right))$  7:  path.pop(0)  8: **end while**  9: reverse( π )10: **return** π

**Proof for the data sample.** The Proof Generation algorithm only proves that the classification result $y_{a}$ is obtained from the decision tree prediction. However, the verification is not fully guaranteed since the client does not know whether the returned result is the classification of the data sample a or not. Thus, CS should provide an extra proof to convince the client that the results are indeed calculated from the input data. We aim to collect all the attributes used in the prediction and check whether the data sample a contains those attributes. CS generates a vector $\bar{a}$ ordered by the decision sequences of the attributes, i.e., v.in of all nodes on the prediction path. However, the arrangement of attributes used and collected in our VDT scheme differs from the attribute arrangement of input data a. Therefore, we use a permutation test to verify that $\bar{a}$ is indeed a rearrangement of the input data a. For a prediction path with *d* categorical attributes, CS generates the permutation index pair according to the order:

$$\bar{a}=\left(path\left[0\right].id,\bar{a}\left[path\left[0\right].id\right]\right),\ldots ,\left(path\left[d-1\right].id,\bar{a}\left[path\left[d-1\right].id\right]\right).$$

CS then sends $\bar{a}$ to the client for verification.

### 4.4. Result Verification

After receiving the inference result and related proofs from CS, the client checks the decision tree’s consistency. The user first invokes the smart contract to obtain the hash value of the decision tree root node stored on the blockchain. Given the random number *r*, the client opens the commitment and compares it to the ADS root hash stored on the blockchain. If they match, the client believes the decision tree has not changed.

**Verification for prediction path.** Then, the client checks the verification of the prediction path. Algorithm 4 implements the specific Prediction Path Verification algorithm to verify the correctness of the prediction path. Since our decision tree model is a binary decision tree, all hash calculations involve two elements. The first and second items in π are the hash values of the leaf nodes, and the last item is the hash value of the root node of the decision tree. To be concrete, the algorithm starts from the first two elements in the prediction path π and iteratively calculates the hash value of the concatenation of the two elements until all the elements in π are involved in the calculation (lines 2–4). After that, the calculation result is compared to the root hash value sent from the blockchain (lines 5–8). If they are equal, the verification passes; otherwise, it fails. The algorithm verifies that the prediction path exists in the ADS structure.
**Algorithm 4:** Prediction Path Verification**Input:** Verification Proof π, Hash value stored on the blockchain $hash_{root}$**Onput:** 0,11: Initialize $hash_{cal}:=\pi \left[0\right]$2: **for **i=1 to N−2 **do**3: **    **$hash_{cal}=H(hash_{cal}\left||\pi \left[i\right]\right)$4: **end for**5: **if **$hash_{cal}=hash_{root}$ **then**6: **    return** 1 for verification passed7: **else**8: **    return** 0 for verification failed9: **end if**

    Another security concern for validating the prediction path is that the client needs to learn whether each decision step works appropriately. Thus, the client needs to check that for each node $v_{i}$ in the prediction path whose order in $\bar{a}$ is *j*, it holds that (1) $v_{i}.in=i$; and (2) $v_{i+1}=v_{i}.left$ if $\bar{a}\left[i\right]<v_{i}.thr$, otherwise $\bar{a}\left[i\right]>v_{i}.thr$. With the help of the construction of $\bar{a}$ and the existing method for equality tests and comparisons in [31], this concern can be efficiently solved by using an arithmetic circuit.

**Verification for data sample.** The verification of the data sample ensures that the result $y_{a}$ is the accurate classification of data sample a. For a decision tree with *d* categorical attributes, the input data a can be expressed as (1,a[1]),…,(d,a[d]). This verification is converted into a permutation test between $\bar{a}$ and a. We adopt the characteristic polynomial technique proposed in [32]. For example, given a vector $c=(c\left[1\right],\ldots ,c\left[d\right]\in F^{d}$, the characteristic polynomial of the vector c is $\chi_{c}\left(x\right)$ = $\prod_{i=1}^{d}\left(x-c\left[i\right]\right)$. Thus, the permutation test transforms into the proof that the characteristic polynomial of c and $\bar{c}$ have the same value at a random point r selected in the field *F*, that is:

(1) $$\prod_{i=1}^{d}\left(r-c\left[i\right]\right)=\prod_{i=1}^{d}\left(r-\bar{c}\left[i\right]\right).$$

For the proof of permutation test of index pairs, each index pair is first packed into a single value using random linear combinations. For each index pair (*i*,a[i]) and (*i*,$\bar{a}\left[i\right]$), by selecting random number *z*, the packed value is c[j]=a[i]+z∗i and $\bar{a}\left[i\right]+z*i$, respectively. Then, the client selects a random number *r* to complete the permutation test. If the permutation test passes, the verification of the data sample is completed. Otherwise, the verification failed.

Together with the commitment and the prediction path verification, the result verification process ensures that $y_{a}$ is the correct classification result of a. The client accepts the result only if all the above verification passes.

## 5. Verifiable Decision Tree Update

The above section solves the verifiable problem of decision tree prediction, and this section focuses on a practical approach.In the real world, decision tree classifiers may retrain and update the model based on the situations and classification tasks. To maintain the verifiability of our VDT scheme and adapt to scenarios where the decision tree model changes, a naive approach is to construct the ADS structure for the new decision tree model. However, we have observed that when the classification task has few changes, the decision tree changes little. Inspired by this phenomenon and the node insertion and update technique in the Merkle tree, we design verifiable decision tree update algorithms for decision tree classifiers with minor dynamic updates. After the decision tree update process, our VDT scheme can be directly used to provide the verifiability for the new decision tree prediction process without additional modification. Our update algorithms consist of three steps:

(1) CS trains a new decision tree classifier; here, we assume that there are few differences between the prior and new models.
(2) CS reconstructs the ADS of the prior model and generate proofs for the differences between the two model.
(3) The blockchain receives the proof for update and verifies the completeness of the updated proof by a smart contract. After the verification is passed, a new hash value of the root node is calculated and stored on the blockchain.

Since there are few changes in the decision tree, the updated proof provided by CS to the blockchain only needs to include the difference between the prior and new decision trees, i.e., the information of the modified nodes. Considering that most nodes make up changed subtrees, we decompose the update operation of the decision tree to the deletion and insertion of subtrees on the decision tree model. The apparent problem with updating decision trees on the blockchain is that the blockchain needs the structure of a complete decision tree model. The blockchain requires the CS to send the proof information to update the hash value of the root node securely. Thus, CS needs to provide an updated proof for all the changed subtrees. CS first finds all *m* changed subtrees ($T_{s_{1}}$,…,$T_{s_{m}}$) by traversing the difference between the two trees and the path from the root node of the decision tree to each subtree’s root node $path_{i}$. Secondly, CS locates the root nodes of all the subtrees and records a piece of location information for each node. In our binary decision tree setting, each internal node has at most two children, and the threshold of the left child node is smaller than the threshold of the right child node. For each decision branch of a node, we define the pointer to the left children as 0 and the pointer to the right children as 1. Thus, the location information inf of the root nodes of all the subtrees can be represented as a binary bit string up to *h* bits, where *h* is the tree’s height.

**Figure 4:**
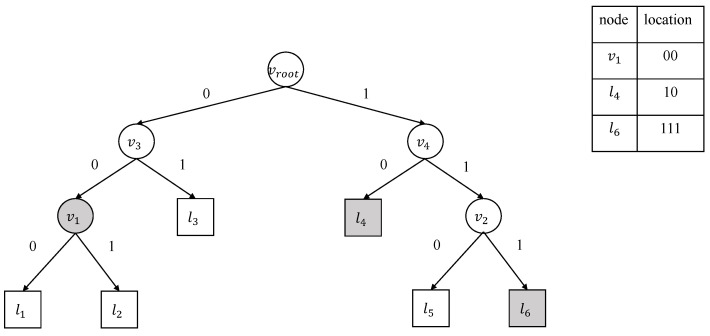
A toy example query of location information denoting the shaded nodes’ location information.

Figure 4 denotes the query of location information. For example, the location information of node $v_{1}$, $l_{4}$, and $l_{6}$ (shaded in Figure 4) is the bit string 00, 10, and 111, respectively. After that, CS constructs the ADS structure for all the subtrees and updates the hash value using the hash function *H*. Meanwhile, CS records the update operation of each subtree insertion or deletion using the bit string *b*, with insertion being 1 and deletion being 0. Algorithm 5 generates the updated proof. The algorithm takes the updated decision tree model and the subtree information as input and constructs updated proofs for each subtree. Since there are *m* subtrees, the final updated proof is $UP={\left[UP_{i}\right]}_{i=1}^{m}$. The updated proof consists of the location information inf, the updated proof for subtrees, the hash value of the root of the decision tree, and the bit string *b*.
**Algorithm 5:** Updated Proof (CS)**Input:** Update decision tree model $T^{\prime }$, the root nodes of the subtrees $T_{i}$, the path of the subtree $path_{i}$**Onput:** Updated Proof $UP_{i}$  1: Initialize $curnode=T^{\prime }$  2: **for **i=1 to *m*  3:     Find the path $path_{i}$ from root of the entire tree to $T_{i}$  4: **    while **curnode is not $T_{i}$ **do**  5: **        **$UP_{i}$.append(the other child node of the curnode not on $path_{i}$)  6: **        **curnode← the child node of curnode on $path_{i}$  7: **    end while**  8: **    if **curnode is $T_{i}$ **then**  9: **        **$UP_{i}$.append($T_{i}$)10: **    end if**11: **end for**12: **return** $UP_{i}$

After receiving the updated proof from CS, the blockchain first confirms the location of each subtree by inf. Then, by invoking a smart contract, the blockchain calculates the hash value of the root node of each subtree from the leaf node to the root node. Specifically, keeping the hash values of other nodes unchanged, the smart contract recalculates the hash value of the ADS root node. When the reconstructed root hash value is the same as the hash value of the root of the ADS, the correctness of the update is verified. It means that the hash value of other nodes has not changed, and the subtrees are the part newly added to the decision tree model. Finally, the smart contract stores the updated version of the decision tree and updates the root hash on-chain.

## 6. Security Analysis

In this section, we analyze the security of the proposed VDT algorithms. As mentioned in Section 3.2 and Section 3.3, we mainly analyze the soundness and completeness of the overall scheme.

**Theorem** **1.** *Our VDT scheme satisfies soundness and completeness if the hash function has the properties of irreversible and collision-resistance, and the executions on the blockchain are secure.*

**Proof.**  This theorem is true when all three processes of the entire verification process (see Section 4.3) satisfy soundness and completeness. The commitment $com_{T}$ guarantees the completeness of the scheme straightforwardly. Since the hash function establishes the commitment, the soundness holds due to the collision-resistance property of the hash function.

Next, we prove the correctness of prediction path verification. Recall that the client verifies the prediction path verification by comparing the calculated hash value based on the proof provided by the CS and the root hash value stored by the blockchain. The prove π contains the hash value of the leaf node representing the prediction result and the hash value of all root nodes of the prediction path. Therefore, based on the binary verifiable decision tree construction, $hash_{cal}$ can be obtained by calculating every two elements starting from the first item. Therefore, if the prediction result has not been tampered with, we have $hash_{cal}=hash_{root}$, which stands for the completeness of the algorithm. Since our scheme is constructed based on hash values and Merkle tree structure, the soundness of the algorithm is based on the collision-resistance property of the hash function. As long as the collision-resistance property of the hash function remains unchanged, our VDT scheme is correct. Since only the nodes’ hash values are used in the proof process, clients cannot obtain the plaintext of the decision tree model during the verification process.

Finally, we prove the soundness and completeness of the permutation test performed in Section 4.4. The completeness of the permutation test can be derived from the characteristic polynomial. If $\bar{a}$ is the permutation of a, then Formula (1) always holds regardless of the random point *r*. The client can conclude that the result is calculated from data a.

As for the soundness of the permutation, suppose that $\bar{a}$ is not a permutation of a, then there must be an index pair (i,a[i]) that is not included in $\bar{a}$. According to the random linear combination, we have:

$$Pr\left[a\left[i\right]+z*i=\bar{a}\left[i\right]+z*i|\left(i,a\left[i\right]\right)\neq \left(i,\bar{a}\left[i\right]\right)\right]\leq \frac{1}{\left|F\right|})].$$

The probability that there exists an index pair (*i*, $\bar{a}\left[i\right]$) with the same result as the random linear combination of (i,a[i]) in $\bar{a}$ is less than $\frac{1}{\left|F\right|}$. Therefore, the probability that the characteristic polynomial *c* and $\bar{c}$ have the same root is less than or equal to $\frac{d}{\left|F\right|}$, and the soundness error is less than $\frac{2d}{\left|F\right|}$. Since d≪F, the soundness of the permutation test is guaranteed. □

**Theorem** **2.** *Our Verifiable Decision Tree Update algorithm is secure if the underlying cryptographic primitives and the executions on the blockchain are secure.*

**Proof.**  Recall that the update algorithm consists of inserting and deleting nodes, and the related proofs are provided by UP. A successful attack would mean either the executions on the blockchain are not secure or the input information is tampered with. The first case is a contradiction to the assumption. For the second case, a tampered proof $UP^{\prime }$ that passes the verification means that there exist two decision trees with different nodes but the same root hash. However, this implies a collision of the hash function, which contradicts the security assumption of underlying cryptographic primitives. Thus, our update algorithm is secure. □

**Formal verification of the smart contract.** Since our scheme applies the smart contract to achieve interaction between blockchain and other entities, we use a formal verification method [33] to verify the correctness of the smart contract to prevent errors and security breaches. Following the idea proposed in [34], we use the BIP (Behavior Interaction Priorities) framework for smart contract verification [35]. Atomic components are used to describe the system behavior, and the obtained smart contract behavior model is essentially a finite-state machine. Then, we use SMC (Statistical Model Checking) tool to check whether the behavior model satisfies the safeness of the system. The formal verification of the smart contract guarantees the integrity of the smart contract executed on the blockchain.

## 7. Experiments and Evaluations

In this section, we evaluate the performance of the proposed verifiable decision tree scheme and the update algorithm.

### 7.1. Experiment Setup

In our experiments, we simulate all phases of our scheme on a Windows 11 computer. Since our implementation is not parallelized for the two entities in the scheme, we only use a single CPU core in the experiments. We execute the experiments ten times and calculate the average execution time as the results. The scheme is implemented in Python.

We use Geth to deploy a private Ethereum network and choose SHA-256 as the cryptographic hash function in all algorithms. We use the Breast Cancer Wisconsin (Original) and Wine Quality datasets from the UCI machine learning repository datasets [36] and train the decision tree using the scikit-learn package in Python. Breast Cancer Wisconsin (Original) is a dataset used for breast cancer diagnosis, which is usually used in machine learning diagnosis scenarios [4]. Each sample contains 10 attributes, and the total number of data samples we used is 400. The pre-trained decision tree $T_{1}$ has 8 levels and 55 nodes. The larger dataset is the Wine Quality dataset, used for the quality evaluation of wine. Each sample contains 12 attributes, and the total number of data samples we used is 1000. The pre-trained decision tree $T_{2}$ has 12 levels and 156 nodes. Table 2 shows the statistical information of the decision tree.

### 7.2. Performance of VDT

We evaluated the performance of the VDT scheme according to the system workflow. The following metrics are measured and reported: (1) the model initialization time $t_{1}$; (2) the proof construction time $t_{2}$ and the overall proof size *s*; and (3) the result verification time $t_{3}$. Among all the parameters, $t_{1}$ remains unchanged when inputting different data samples. For decision trees $T_{1}$ and $T_{2}$, the model initialization time is 3.21 ms and 16.44 ms, respectively. As we can see, although the model initialization time of VDT increases rapidly with the growth of nodes and height, the initialization time is only in the order of milliseconds. This is because the preprocessing in our VDT only involves computing hashes and tree construction, which is very fast in practice. Specifically, since we focus on binary decision trees with the height *h*, the maximum number of hash calculations required to build ADS is $2^{h}-1$.

To evaluate $t_{2}$, $t_{3}$, and *s*, we vary the length of the prediction path lp from 6 to 12. The prediction of the first 2 columns is chosen in decision tree $T_{1}$, and the last 2 columns are obtained from decision tree $T_{2}$. Table 3 denotes our scheme’s time and computation cost. As shown in Table 3, our scheme is effective and has practical application value. The construction time for generating a proof of a predicted path with a length of 12 is 102.4 ms, and the proof size is 1.12 KB. Moreover, the proof construction time and the result verification time scales roughly linearly with the length of the prediction path of the decision trees, while the attributes and the difference between $T_{1}$ and $T_{2}$ have minimal impact. This aligns with our expectations because we do not need to consider other attributes except tree height in implementing our VDT scheme.

**Figure 5:**
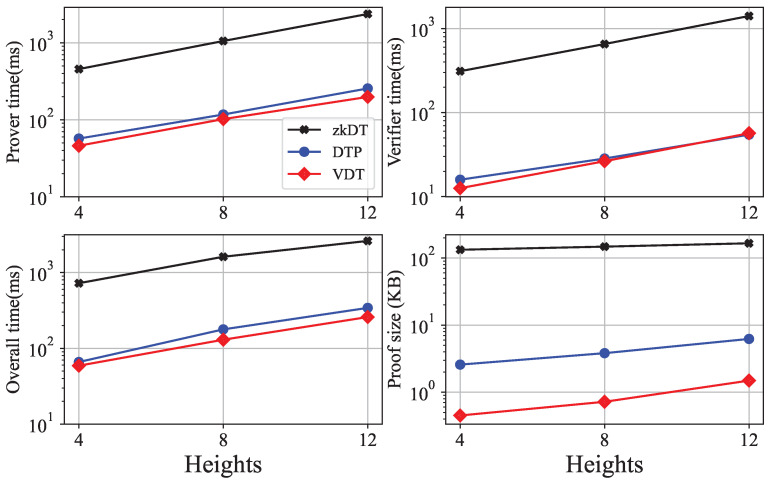
Performance of the three schemes.

Then, we compare our scheme with other verifiable decision tree prediction schemes, i.e., a zero knowledge decision tree prediction scheme [24] using zero-knowledge proofs and a public verifiable private decision tree prediction scheme [25] using non-interact zero-knowledge proofs. For convenience, we call them zkDT and DTP, respectively. We have prepared four well-trained decision tree models and used VDT, zkDT, and DTP, respectively, to realize verifiable predictions of decision tree models. Then, we record the running time of using the same decision tree to predict the same input data and generate verification proofs to evaluate the efficiency of the schemes. We do not evaluate the consumption of blockchain because the scheme only stores the hash value of the root node of the decision tree on the blockchain, which leads to a minimal storage cost. Figure 5 shows the performance of the three schemes. We evaluate the three schemes on the decision tree with heights of 4, 8 and 16. The running time of our VDT scheme is slightly faster than the DTP scheme and outperforms the zkDT scheme. Our proof sizes are around 1 KB, which is at least 4× smaller than the other schemes. It is because both zkDT and DTP use zero-knowledge proofs, which require more proof parameters than our method.

### 7.3. Model Update Algorithm

For the decision tree update algorithm, we imitate the update by modifying the subtrees in decision tree $T_{2}$. We evaluate the influence of dynamic update optimization on the computation time and the proof size. We keep the height of the decision tree at 12. We vary the changes of the decision tree by modifying one to four subtrees with a height of 4. We take direct submission as the baseline and compare it with the decision tree update scheme. Table 4 shows the performance results. All hash values need to be recalculated for the baseline method each time, and the calculation time is 27.97 ms. The computation time of the update algorithm is approximately 4–15× faster than the baseline. Moreover, the scheme’s computation time and update size are linearly related to the numbers of the subtree changes. Since the cost of our approach is in the order of milliseconds and kilobytes, the efficiency of our update optimization is reasonable in practice.

### 7.4. Summary and Application of Our Scheme

We proposed a verifiable decision tree scheme named VDT to ensure the integrity and correctness of decision tree prediction results. By leveraging the hash function and the Merkle tree, we construct an ADS structure to provide verification proofs. By storing the relevant information of the decision tree on the blockchain, we have implemented an efficient and reliable prediction verification scheme on a hybrid blockchain system. We further extend our scheme to support decision tree updates. Theoretical analysis shows that our scheme guarantees soundness and completeness. Experimental evaluation shows that our scheme requires less than one second to produce verifiable proofs, outperforming existing work regarding efficiency. Meanwhile, the proof sizes are around 1 KB, which is at least 4× smaller than the other schemes.

Furthermore, our verifiable decision tree scheme can also deploy homomorphic encryption mechanisms, as discussed in [37,38], to build a fair and secure machine learning training and predicting platform on the hybrid blockchain system [39,40]. Machine learning models are personal private properties. Model owners hope to realize the agency or sales of machine learning models to obtain remuneration. Meanwhile, blockchain is a gradually rising technique in which all nodes can verify the data, which is valid and tamper-proof data in the block. Applying blockchain in the machine learning platform to realize the hybrid blockchain system of blockchain and cloud servers can achieve fair and complete services without a trusted party. The smart contract can ensure the simultaneous payment and delivery of the data execution protocol. Our verifiable decision tree scheme in this paper can be used to create a fair and secure machine learning training and predicting platform with the help of blockchain and smart contracts. Realizing the system of this machine learning platform is left as future work.

## 8. Related Work

### 8.1. Verifiable Outsourced Computation

Verifiable computation is universally used to verify the correctness of outsourced computation tasks to apply outsourced computing resources. Various cryptographic methods, e.g., interactive proofs (IPs) [41], succinct non-interactive arguments of knowledge (SNARK) [42,43], and Authenticated Data Structure (ADS) [44] have become the mainstream methods of constructing verifiable outsourced computations to provide computational integrity. IPs are implemented according to the sum-check protocol and are often converted to circuits for proof. For example, Goldwasser et al. [41] proposed an interactive GKR protocol to generate a general proof for outsourced computation by representing the computation as a layered arithmetic circuit and then using the sum-check protocol to ensure the correctness of the arithmetic circuit. However, converting a verifiable outsourced computation task to a circuit remains challenging as it requires vast computation. Meanwhile, these IPs require interaction between the verifier and prover, which suffers huge communication costs. SNARKs can transform an arbitrary polynomial task into a circuit to produce a brief proof. Gennaro et al. [45] proposed a zk-SNARK scheme based on the quadratic arithmetic program (QAP). QAP allows the transformation between arithmetic circuits and polynomial equations, while zero-knowledge proofs [41] guarantee the integrity of the whole scheme. Nevertheless, the computation cost of SNARK remains large because of the complexity of the circuit-based approach and the zero-knowledge proofs. In contrast, ADS is simple in structure and can be combined with other cryptography technologies to guarantee the privacy and integrity of the verifiable outsourced computation tasks.

### 8.2. Verifiable Machine Learning

Utilizing machine learning prediction services on the cloud to analyze data samples efficiently has increased attention. Verifiable machine learning prediction schemes for various models are proposed to ensure the inference process is correctly performed. Many verifiable machine learning systems are proposed to solve the problem of verifiability in the inference stage of different machine learning models. In [46], Ghodsi et al. proposed a system named SafetyNet to construct a scheme that enables the untrusted cloud server to generate a short mathematical proof to let clients verify the correctness of inference tasks they raised. Lee et al. [19] proposed a verifiable scheme for CNN prediction named vCNN, where users can verify the validity of the predicted values of the CNN model without the weights and the third party without data and weights. In concrete, the scheme used a quadratic arithmetic program technique and commit-and-proof method to generalize the verification of CNN in a zero knowledge way. Some enhanced schemes, such as [20], are then presented to improve the efficiency of [19]. Feng et al. [18] proposed ZEN, an optimizing compiler that generates a zero-knowledge proof for the CNN inference scheme. ZEN draws the optimization of a quantization algorithm and stranded encoding to develop rapid proof for CNN inference. Liu et al. [21] initiated the study of zero knowledge convolutional neural networks and proposed a zkCNN scheme for verifying CNN prediction and accuracy. Specifically, the authors design a new sum check protocol for CNN and generalize the GKR protocol to design the zero-knowledge CNN. However, these prior works utilize zero-knowledge proof to process the verification. As such, they suffer from substantial computational costs. Our VDT scheme provides an efficient way for verifiable decision tree predictions.

## 9. Conclusions

In this paper, we have studied the problem of verifying the integrity of the decision tree models in the cloud service system. To reduce the computation cost of verification proof, we have proposed the Merkle-tree-based verifiable decision tree structure that incurs a small computational overhead to generate while supporting efficient verification of the prediction results on the client side. Furthermore, according to the scene’s needs, we designed a verifiable update method for the decision tree to reduce the overhead of updating the information stored on the blockchain. Theoretical analysis and empirical results have validated the proposed techniques’ correctness, security, and efficiency.

Our proposed verifiable decision tree scheme can be easily extended to other tree-structured machine learning models, such as multivariate decision trees and random forest algorithms. For future work, we are interested in exploring the verification of other machine learning training and inference algorithms on hybrid blockchain systems. This research field poses challenges since no generic verifiable approach is designed for all machine learning algorithms in the cloud-serving system to ensure the integrity of the prediction result and privacy security.

## Figures and Tables

**Table 1:** Notations used in our scheme.

Notation	Description
*T*	The authenticated data structure constructed on the decision tree
*h*	The height of the decision tree
*N*	The number of nodes in the decision tree
a	The data sample
$\bar{a}$	The permutation of data sample
*v*	The internal node of the decision tree
*l*	The leaf node of the decision tree
v.in	The associate attribute index of an internal node *v*
v.thr	The threshold of an internal node *v*
v.left	The left child node of parent node *v*
v.right	The right child node of parent node *v*
l.class	The classification result of the leaf node *c*
*H*	The hash function
hash	The hash value
$y_{a}$	The classification result of the data sample

**Table 2:** Statistical information of the decision tree.

Decision Tree	Number of Attributes	Number of Data Samples	Height *h*	Number of Nodes *N*
$T_{1}$	10	400	8	55
$T_{2}$	12	1000	12	156

**Table 3:** Evaluation of the VDT scheme.

**Length** lp	6	8	10	12
**Proof construction time** $t_{2}$** (ms)**	46.2	59.8	81.6	102.4
**Result verification time** $t_{3}$ **(ms)**	19.5	26.4	36.1	42.3
**Overall proof size** *s*** (KB)**	0.63	0.72	0.97	1.12

**Table 4:** Evaluation of model update algorithm.

**Subtree changes**	1	2	3	4
**Base.time (ms)**	47.97			
**Upt.time (ms)**	3.28	6.09	9.57	12.59
**Base.size (KB)**	2.19			
**Upt.size (KB)**	0.45	0.90	1.35	1.8

## Data Availability

This study analyzed publicly available datasets from the UCI Machine Learning Repository. These data can be found here: https://archive.ics.uci.edu/ml/index.php, (accessed on 14 March 2023).

## Abbreviations

The following abbreviations are used in this manuscript:

ADS	Authenticated Data Structure
CS	Cloud server

## References

[1] Lecun Y., Bottou L., Bengio Y., Haffner P. (1998). Gradient-based learning applied to document recognition. Proc. IEEE.

[2] Hedderich M.A., Lange L., Adel H., Strötgen J., Klakow D. (2020). A Survey on Recent Approaches for Natural Language Processing in Low-Resource Scenarios. arXiv.

[3] Xu L., Wang Z., Wu B., Lui S. MDAN: Multi-level Dependent Attention Network for Visual Emotion Analysis. Proceedings of the 2022 IEEE/CVF Conference on Computer Vision and Pattern Recognition (CVPR).

[4] Afolayan J.O., Adebiyi M.O., Arowolo M.O., Chakraborty C., Adebiyi A.A., Chakraborty C., Khosravi M.R. (2022). Breast Cancer Detection Using Particle Swarm Optimization and Decision Tree Machine Learning Technique. Intelligent Healthcare: Infrastructure, Algorithms and Management.

[5] Wu Y., Wang X., Susilo W., Yang G., Jiang Z.L., Chen Q., Xu P. (2021). Efficient Server-Aided Secure Two-Party Computation in Heterogeneous Mobile Cloud Computing. IEEE Trans. Dependable Secur. Comput..

[6] Weng J., Weng J., Cai C., Huang H., Wang C. (2022). Golden Grain: Building a Secure and Decentralized Model Marketplace for MLaaS. IEEE Trans. Dependable Secur. Comput..

[7] Zhang C., Xu C., Wang H., Xu J., Choi B. Authenticated Keyword Search in Scalable Hybrid-Storage Blockchains. Proceedings of the 2021 IEEE 37th International Conference on Data Engineering (ICDE).

[8] Ge Z., Loghin D., Ooi B.C., Ruan P., Wang T. (2022). Hybrid Blockchain Database Systems: Design and Performance. Proc. VLDB Endow..

[9] Zhang C., Zhao M., Zhu L., Zhang W., Wu T., Ni J. (2022). FRUIT: A Blockchain-Based Efficient and Privacy-Preserving Quality-Aware Incentive Scheme. IEEE J. Sel. Areas Commun..

[10] Fredrikson M., Jha S., Ristenpart T. (2015). Model Inversion Attacks That Exploit Confidence Information and Basic Countermeasures. Proceedings of the 22nd ACM SIGSAC Conference on Computer and Communications Security, Denver, CO, USA, 12–16 October 2015.

[11] Nasr M., Shokri R., Houmansadr A. (2018). Machine Learning with Membership Privacy using Adversarial Regularization. Proceedings of the 2018 ACM SIGSAC Conference on Computer and Communications Security.

[12] Li W., Xiang L., Zhou Z., Peng F. Privacy Budgeting for Growing Machine Learning Datasets. Proceedings of the IEEE INFOCOM 2021—IEEE Conference on Computer Communications.

[13] Zhang C., Hu C., Wu T., Zhu L., Liu X. (2022). Achieving Efficient and Privacy-Preserving Neural Network Training and Prediction in Cloud Environments. IEEE Trans. Dependable Secur. Comput..

[14] Wu Y., Wang X., Susilo W., Yang G., Jiang Z.L., Li J., Liu X. (2022). Mixed-protocol multi-party computation framework towards complex computation tasks with malicious security. Comput. Stand. Interfaces.

[15] Horchulhack P., Kugler Viegas E., Santin A. (2021). Toward feasible machine learning model updates in network-based intrusion detection. Comput. Netw..

[16] Mohassel P., Zhang Y. SecureML: A System for Scalable Privacy-Preserving Machine Learning. Proceedings of the 2017 IEEE Symposium on Security and Privacy (SP).

[17] Niu C., Wu F., Tang S., Ma S., Chen G. (2022). Toward Verifiable and Privacy Preserving Machine Learning Prediction. IEEE Trans. Dependable Secur. Comput..

[18] Feng B., Qin L., Zhang Z., Ding Y., Chu S. (2021). ZEN: An Optimizing Compiler for Verifiable, Zero-Knowledge Neural Network Inferences. Cryptol. Eprint Arch..

[19] Lee S., Ko H., Kim J., Oh H. (2020). vCNN: Verifiable Convolutional Neural Network. IACR Cryptol. Eprint Arch..

[20] Weng J., Weng J., Tang G., Yang A., Li M., Liu J.N. (2022). pvCNN: Privacy-Preserving and Verifiable Convolutional Neural Network Testing. arXiv.

[21] Liu T., Xie X., Zhang Y. (2021). ZkCNN: Zero Knowledge Proofs for Convolutional Neural Network Predictions and Accuracy. Proceedings of the 2021 ACM SIGSAC Conference on Computer and Communications Security.

[22] Li X., He J., Vijayakumar P., Zhang X., Chang V. (2022). A Verifiable Privacy-Preserving Machine Learning Prediction Scheme for Edge-Enhanced HCPSs. IEEE Trans. Ind. Inform..

[23] Hu C., Zhang C., Lei D., Wu T., Liu X., Zhu L. (2023). Achieving Privacy-Preserving and Verifiable Support Vector Machine Training in the Cloud. IEEE Trans. Inf. Forensics Secur..

[24] Zhang J., Fang Z., Zhang Y., Song D. (2020). Zero Knowledge Proofs for Decision Tree Predictions and Accuracy. Proceedings of the 2020 ACM SIGSAC Conference on Computer and Communications Security.

[25] Wang H., Deng Y., Xie X. (2020). Public Verifiable Private Decision Tree Prediction. Proceedings of the Information Security and Cryptology: 16th International Conference, Inscrypt 2020.

[26] Quinlan J.R. (2004). Induction of Decision Trees. Mach. Learn..

[27] Adebiyi M., Adebiyi A.A., OKesola J.O., Arowolo M.O. (2020). ICA Learning Approach for Predicting RNA-Seq Data Using KNN and Decision Tree Classifiers. Int. J. Adv. Sci. Technol..

[28] Merkle R.C., Brassard G. (1990). A Certified Digital Signature. Proceedings of the Advances in Cryptology—CRYPTO’ 89 Proceedings.

[29] Nakamoto S. (2009). Bitcoin: A Peer-to-Peer Electronic Cash System. https://metzdowd.com.

[30] Szabo N. (1997). Formalizing and Securing Relationships on Public Networks. First Monday.

[31] Parno B., Howell J., Gentry C., Raykova M. Pinocchio: Nearly Practical Verifiable Computation. Proceedings of the 2013 IEEE Symposium on Security and Privacy.

[32] Pesarin F., Salmaso L. (2010). The permutation testing approach: A review. Statistica.

[33] Krichen M., Lahami M., Al-Haija Q.A. Formal Methods for the Verification of Smart Contracts: A Review. Proceedings of the 2022 15th International Conference on Security of Information and Networks (SIN).

[34] Abdellatif T., Brousmiche K.L. Formal Verification of Smart Contracts Based on Users and Blockchain Behaviors Models. Proceedings of the 2018 9th IFIP International Conference on New Technologies, Mobility and Security (NTMS).

[35] Basu A., Bozga M., Sifakis J. Modeling Heterogeneous Real-time Components in BIP. Proceedings of the ICSC Congress on Computational Intelligence.

[36] Dua D., Graff C. (2017). UCI Machine Learning Repository. http://archive.ics.uci.edu/ml.

[37] Liu L., Chen R., Liu X., Su J., Qiao L. (2020). Towards Practical Privacy-Preserving Decision Tree Training and Evaluation in the Cloud. IEEE Trans. Inf. Forensics Secur..

[38] Akavia A., Leibovich M., Resheff Y.S., Ron R., Shahar M., Vald M. (2022). Privacy-Preserving Decision Trees Training and Prediction. ACM Trans. Priv. Secur..

[39] Lan Y., Liu Y., Li B., Miao C. Proof of Learning (PoLe): Empowering Machine Learning with Consensus Building on Blockchains. Proceedings of the AAAI Conference on Artificial Intelligence.

[40] Dibaei M., Zheng X., Xia Y., Xu X., Jolfaei A., Bashir A.K., Tariq U., Yu D., Vasilakos A.V. (2022). Investigating the Prospect of Leveraging Blockchain and Machine Learning to Secure Vehicular Networks: A Survey. IEEE Trans. Intell. Transp. Syst..

[41] Goldwasser S., Micali S., Rackoff C. (1985). The Knowledge Complexity of Interactive Proof-Systems. Proceedings of the Seventeenth Annual ACM Symposium on Theory of Computing.

[42] Ames S., Hazay C., Ishai Y., Venkitasubramaniam M. (2017). Ligero: Lightweight Sublinear Arguments Without a Trusted Setup. Proceedings of the 2017 ACM SIGSAC Conference on Computer and Communications Security.

[43] Campanelli M., Fiore D., Querol A. (2019). LegoSNARK: Modular Design and Composition of Succinct Zero-Knowledge Proofs. Proceedings of the 2019 ACM SIGSAC Conference on Computer and Communications Security.

[44] Tamassia R., Di Battista G., Zwick U. (2003). Authenticated Data Structures. Proceedings of the Algorithms—ESA 2003.

[45] Gennaro R., Gentry C., Parno B., Raykova M., Johansson T., Nguyen P.Q. (2013). Quadratic Span Programs and Succinct NIZKs without PCPs. Proceedings of the Advances in Cryptology—EUROCRYPT 2013.

[46] Ghodsi Z., Gu T., Garg S. (2017). SafetyNets: Verifiable Execution of Deep Neural Networks on an Untrusted Cloud. Proceedings of the 31st International Conference on Neural Information Processing Systems.

